# 5-Aza-2’-deoxycytidine enhances lipopolysaccharide-induced inflammatory cytokine expression in human dental pulp cells by regulating TRAF6 methylation

**DOI:** 10.1080/21655979.2019.1621135

**Published:** 2019-06-03

**Authors:** Zhihui Feng, Minkang Zhan, Runsha Meng, Xinxuan Wang, Qiong Xu

**Affiliations:** Guanghua School of Stomatology & Guangdong Provincial Key Laboratory of Stomatology, Sun Yat-sen University, Guangzhou, China

**Keywords:** 5-Aza-2’-deoxycytidine, DNA methylation, dental pulp cells, lipopolysaccharide, TRAF6

## Abstract

Dental pulp inflammation is a common bacterially driven inflammation characterized by the local accumulation of inflammatory mediators in human dental pulp. DNA methylation is a crucial epigenetic modification that that plays a fundamental role in gene transcription, and its role in inflammation-related diseases has recently attracted attention. However, its role in dental pulp inflammation is poorly understood. This study is aimed to elucidate the role of DNA methylation in lipopolysaccharide (LPS)-induced inflammatory reaction in human dental pulp cells (hDPCs). hDPCs were pretreated with DNA methylation inhibitor 5-aza-2ʹ-deoxycytidine (5-Aza-CdR) and a cytokine antibody array was used to detect LPS-induced cytokine expression. The results indicated that 5-Aza-CdR significantly increased the expression of several pro-inflammatory cytokines in LPS-treated cells, including IL-6, IL-8, GM-CSF, MCP-2 and RANTES. The increased expression levels of IL-6 and IL-8 were further verified by qRT-PCR and ELISA. Furthermore, pretreatment with 5-Aza-CdR resulted in upregulation of p-IKKα/β, p-IκBα, p-p65 and p-ERK in the NK-κB and MAPK pathways. In addition, the 5mC level of the TRAF6 promoter was significantly decreased following 5-Aza-CdR pretreatment in the LPS-stimulated hDPCs. The findings indicate that 5-Aza-CdR significantly enhances the expression of proinflammatory cytokines and activates the NF-κB and MAPK signaling pathways by eliciting a decline in the 5mc level in the TRAF6 promoter in hDPCs, suggesting that DNA methylation may play an important role in dental pulp inflammation. This study highlights the important role of DNA methylation in the immunity defense of dental pulp infection.

## Graphical Abstract

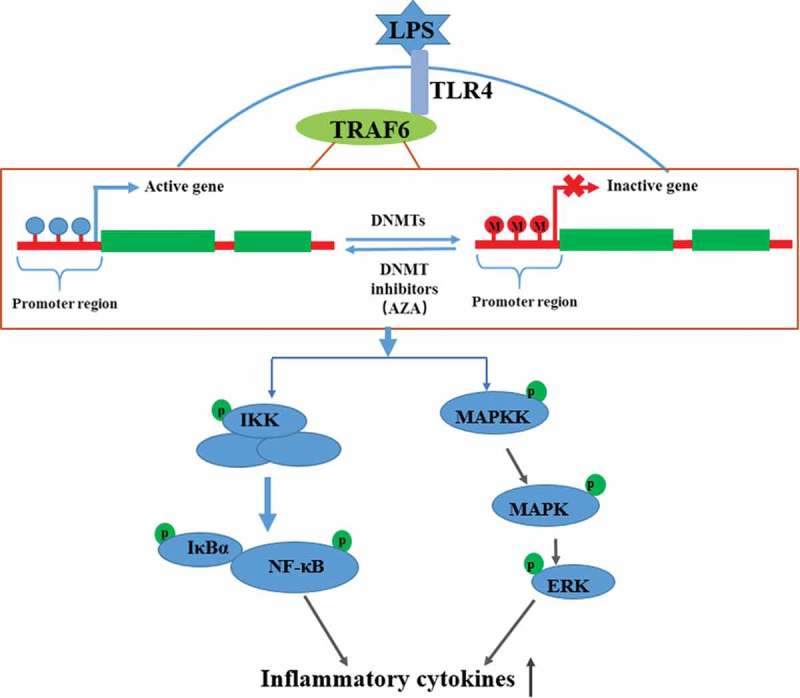

## Introduction

Dental pulp inflammation is a typical inflammation of dental pulp that occurs mainly due to the invasion of bacterial components and byproducts [1]. It often develops into pulp necrosis or periapical disease, which can lead to dental emergencies. Lipopolysaccharide (LPS) is a major component of the outer membrane of gram-negative bacteria. It can be detected in infected dental pulp and periapical lesions and plays key roles in pulpal and periapical diseases [2–5]. LPS can significantly modulate the expression of cytokines and chemokines by activating the TLR4-mediated NF-κB and MAPK signaling pathways in human dental pulp cells (hDPCs) [6,7]. Although many mechanisms are involved in dental pulp inflammation, the specific molecular mechanisms remain unclear. Recent studies have provided evidence indicating that epigenetic events are involved in the development of inflammatory diseases in dental pulp [8–11].

DNA methylation is a crucial epigenetic modification characterized by the addition of a methyl group to the fifth carbon position of the cytosine located in Cytosine-phosphate-Guanine (CpG) dinucleotides in a reaction catalyzed by DNA methyltransferases (DNMTs) [12]. The methylation levels of CpG sites in the gene promoter have been demonstrated to regulate gene expression; a hypermethylated CpG region can inhibit gene transcription, whereas hypomethylation activates gene expression [12,13]. DNA methylation has been recently reported to regulate the inflammatory response and to play important roles in the development of inflammation-related diseases [14]. The hypomethylation of the CpG motif in the promoter of IL-6 in rheumatoid arthritis (RA) is related to the overexpression of IL-6, which may be responsible for the pathogenesis of RA [15]. In airway hyperresponsiveness diseases, the fractional exhaled nitric oxide concentrations were associated with the hypomethylation of the IL-6 and iNOS gene promoter regions in nasal tissue samples from asthmatic children [16]. A study of osteoarthritis (OA) patients demonstrated the key role of DNA methylation status on the expression of IL-8 in human chondrocytes [17]. However, few studies have offered insights into the regulation of DNA methylation in the development of dental pulp inflammation.

The DNMT inhibitor 5-aza-2ʹ-deoxycytidine (5-Aza-CdR) is a cytosine analog that can inhibit the activity of DNMTs and reduce DNA methylation levels by establishing a covalent complex between the DNMTs and 5-Aza-CdR-substituted DNA [18]. 5-Aza-CdR is thus widely used as an epigenetic modulator to demonstrate DNA methylation in cells [19]. This study aimed to investigate the effect of 5-Aza-CdR on LPS-induced inflammatory cytokine expression and activation of the NF-κB and MAPK signaling pathways in hDPCs, thereby exploring the role of DNA methylation in the inflammation of dental pulp. Our results demonstrated that 5-Aza-CdR promoted the expression of several pro-inflammatory cytokines and the phosphorylation of IKKα/β, IκBα, p65 and ERK in LPS-induced hDPCs. Meanwhile, demethylation treatment with 5-Aza-CdR decreased 5mc level in the TRAF6 promoter, an important signal transduction in NF-κB and MAPK signaling pathways. These data suggested that 5-Aza-CdR significantly enhances the inflammatory response of hDPCs by declining the methylation level of TRAF6 promoter.

## Materials and methods

### Isolation and culture of hDPCs

The study was approved by the Ethical Review Board of the Guanghua School of Stomatology of Sun Yat-sen University. All patients who enrolled in the study gave written informed consent for this investigation. The consent forms included the details outlined in the principles of the Declaration of Helsinki. Healthy permanent premolars and third molars were collected from donors aged 18 to 25 years. Only healthy teeth without carious disease or hyperemic pulp tissue were selected. A total of 120 teeth from 56 donors were obtained to isolate the dental pulp tissue and incubate cells. The hDPCs were isolated and cultivated from human dental pulp tissue using an enzymatic method as described by Gronthos et al. [20]. After extraction, the teeth were washed with 70% ethanol and phosphate-buffered saline (PBS, pH 7.4). The teeth were then split open to expose the pulp chamber; the pulp tissue was gently removed with forceps and minced into fragments. The small fragments of minced pulp tissue were digested in a solution with 3 mg/mL type I collagenase and 4 mg/mL dispase for 30 min at 37°C (Gibco, Carlsbad, CA). Following digestion, the pulp tissues were placed in 25 cm^2^ culture flasks containing DMEM (Gibco) containing 10% fetal bovine serum, 100 U/ml penicillin, and 100 mg/ml streptomycin (Gibco) at 37°C with 5% CO_2_. The medium was changed every 3 d. The cells were detached using trypsin/EDTA (Gibco) and subcultured at a ratio of 1:3 when they reached 80% confluence. All experiments were performed with cells from passage 2 to 3.

### Treatment with 5-Aza-CdR and LPS stimulation

Cells were seeded into 6-well plates at the concentration of 5 × 10^4^ per well. 5-Aza-CdR (Sigma, St. Louis, MO, USA) was diluted in DMSO at a dose of 10 mM and used at 10 μM concentration. The dosages and exposure time to 5-Aza-CdR were modeled after previous experiments that demonstrated low cytotoxicity using a cell counting kit (Dojindo, Kumamoto, Japan). According to the different culturing conditions, hDPCs were divided into four groups, as shown in Table 1. After culturing for 24 h, cells were treated with 10 μM 5-Aza-CdR for 48 h to achieve DNA demethylation. Following 5-Aza-CdR pretreatment, cells were washed with PBS and then stimulated with 1 μg/ml *Escherichia coli* LPS (Sigma, USA) for the indicated times. Cells without LPS stimulation or 5-Aza-CdR treatment were used as blank controls.

**Table 1.** Culture conditions of each group.

Groups	Culture medium (48 h)	Culture medium (24 h)
Control	DMEM	DMEM
LPS	DMEM	1 μg/ml LPS
5-Aza-CdR	10 μM 5-Aza-CdR	DMEM
5-Aza-CdR+LPS	10 μM 5-Aza-CdR	1 μg/ml LPS

### Cytokine antibody array and enzyme-linked immunosorbent assay (ELISA)

The cell culture supernatants were collected and spun at 2000 rpm for 20 min to remove cell debris. The detection of cytokines and chemokines in supernatants obtained from different groups was performed using the RayBio® C Series Human Cytokine Antibody Array C3 (RayBiotech, Hercules, USA) in accordance with the manufacturer’s instructions. Briefly, the membrane was blocked with a blocking buffer for 30 min at room temperature. The blocking buffer was then removed and 1 ml of sample supernatant was added to the membrane and incubated for 5 h. After washing 3 times, the membrane was incubated with the antibody cocktail at 4°C overnight and then blocked with HRP-streptavidin for 2 h at room temperature. The protein spots were observed using an ImageQuant LAS 4000 mini system (GE Healthcare Life Sciences, NJ, USA) and normalized to the control spots.

The concentrations of IL-6 and IL-8 in the culture supernatants were analyzed using an ELISA kit according to the manufacturer’s protocol (R&D, Minneapolis, MN, USA). Optical density (OD) values were measured at 450 nm using a microplate reader. Sample concentrations were calculated according to the corresponding OD value and the concentration of the standard substance.

### Real-time quantitative polymerase chain reaction (qRT-PCR)

The total RNA of hDPCs was extracted using TRIzol reagent (Invitrogen, Carlsbad, CA) following the manufacturer’s instructions. Chloroform was added to samples and then centrifuged at 12,000 g for 15 min. The colorless upper aqueous phase containing RNA was transferred to a fresh tube. Isopropyl alcohol was used to precipitate RNA and the RNA pellet was then rinsed with 75% ethanol. The concentration and purity of isolated RNA were determined by NanoDrop 2000c Spectrophotometer (Thermo Fisher Scientific Inc., Waltham, MA, USA). The extracted RNA was treated with RNase-free DNase (Promega, Madison, WI), and 1 μg of RNA from each sample was reverse transcribed for complementary DNA (cDNA) synthesis using the RevertAid^TM^ First Strand cDNA Synthesis Kit (Fermentas, Ontario, Canada). Subsequently, the cDNA was used as a template for PCR. qRT-PCR was performed using a Light Cycler 480 with SYBR green I Master (Roche, Basel, Switzerland). The cycling protocol was as follows: pre-incubation at 95°C for 10 min, followed by 40 cycles of amplification at 95°C for 10 s, annealing at 65°C for 20 s, and extension at 72°C for 30 s. All reactions were performed in triplicate. The measured mRNA levels were normalized to the mRNA level of GAPDH. The primers were synthesized by Invitrogen (Life Technologies) and are listed in Table 2.

**Table 2.** Primers used for the analysis of mRNA levels by qRT-PCR.

Gene	Forward primer	Reverse primer
IL-6 (interleukin-6)	5′TGCAATAACCACCCCTGACC3′	5′AGCTGCGCAGAATGAGATGA3′
IL-8 (interleukin-8)	5′GGTGCAGTTTTGCCAAGGAG3′	5′TTCCTTGGGGTCCAGACA GA3′
MCP-2	5′GGGACTTGCTCAGCCAGATT3′	5′CATCTCTCCTT GGGGTCAGC3′
GM-CSF	5′GGGAGCATGTGAATGCCATC3′	5′GGCT CCTGGAGGTCAAACAT3′
RANTES	5′TTCCTGTATGACTCCCGGCT3′	5′GAAGCCTCCCAAGCTAGGAC3′
GAPDH (D-glyceraldehyde-3-phosphate dehydrogenase)	5′TCTCCTCTGACTTCAACAGCGACA3′	5′CCCTGTTGCTGTAGCCAAATTCGT3′

### Western blot analysis

hDPCs were lysed using RIPA lysis buffer. Total protein was measured using a BCA Protein Assay Kit (Beyotime, Haimen, China). Thirty micrograms of protein was separated by electrophoresis on 8% SDS-polyacrylamide gels and transferred to polyvinylidene fluoride (PVDF) membranes (Millipore, Billerica, MA, USA) with transfer buffer containing 10% methanol. After blocking with TBST containing 5% nonfat milk at room temperature for 1 h, the membranes were probed overnight at 4°C with the primary antibodies (1:2000) including IKKα/β, p-IKKα/β, IκBα, p-IκBα, p65, p-p65, p38, p-p38, ERK1/2, p-ERK1/2, JNK, p-JNK (CST, USA) and GAPDH (Abcam, UK). Subsequently, the membranes were incubated for 1 h with secondary antibodies at a dilution of 1:2000 (CST, USA) at room temperature. After the membranes were thoroughly washed with TBST buffer, bands with target proteins were visualized with enhanced chemiluminescence reagents (Millipore, USA) and observed using an ImageQuant LAS 4000 mini system (GE Healthcare Life Sciences, USA). The blots were quantified and normalized using the ImageJ 1.47 software program (National Institutes of Health, MD, USA).

### Methylated DNA immunoprecipitation (MeDIP) and qRT-PCR

Total DNA was obtained using a tissue DNA isolation kit (Omega Bio-Tek, Norcross, GA, USA) according to the manufacturer’s instructions. NanoDrop 2000c Spectrophotometer was used to assess the concentration, integrity and purity of each DNA sample. DNA (3μg) was sonicated into fragments ranging from 200 to 500 bp using a sonicator (Sonics, Newtown, CT, USA). One microgram of fragmented DNA was denatured to produce single-stranded DNA, and immunoprecipitation was performed overnight at 4°C with 2 μg anti-5mC antibody (ab10805, Abcam). Non-specific human IgG immunoprecipitation (IP) was performed in parallel to the methyl DNA immunoprecipitation as a negative control. DNA-antibody complexes were captured by protein A/G beads (Santa Cruze). Finally, the immunoprecipitated DNA was purified with phenol–chloroform and precipitated with ethanol. The harvested DNA fragments were resuspended in 10 μl Tris buffer (10 mM Tris, pH 8.5), and 1 μl of the DNA was used for real-time PCR detection. All of the primers used for the MeDIP-PCR are listed in Table 3.

**Table 3.** The primers used for MeDIP-PCR.

Gene	Forward primer	Reverse primer
MyD88 (myeloid differentiation primary response 88)	5ʹ TTCGCTCACCGACACAGATG3′	5ʹ GGTCACTGCGGCTGCTCTT3′
TRAF6(TNF-receptor-associated factor-6)	5ʹGCTTACTGTAGCCTTGACTGCC3′	5ʹ GTGGTGCATATCTGTAGTCTCGG3′
IL-6 (interleukin-6)	5ʹ TGGCAGCACAAGGCAAACC3′	5ʹ GCTTCAGCCCACTTAGAGGAGG3′
IL-8 (interleukin-8)	5ʹTAGGAAGTGTGATGACTCAGGTT3′	5ʹ GTCAGAGGAAATTCCACGATT3′

### Statistical analyses

Each experiment was performed in triplicate and was repeated at least three times. All data are shown as the means ± SD. The SPSS 20.0 software package (SPSS Inc., Chicago, USA) was used for the statistical tests. The statistical analysis of the differences between the experimental groups was performed using one-way analysis of variance (ANOVA) or repeated-measures ANOVA. A value of *P* < 0.05 was considered to indicate statistical significance.

## Results

### 5-Aza-CdR stimulated the expression of inflammatory cytokines in LPS-induced hDPCs

**Figure 1.**
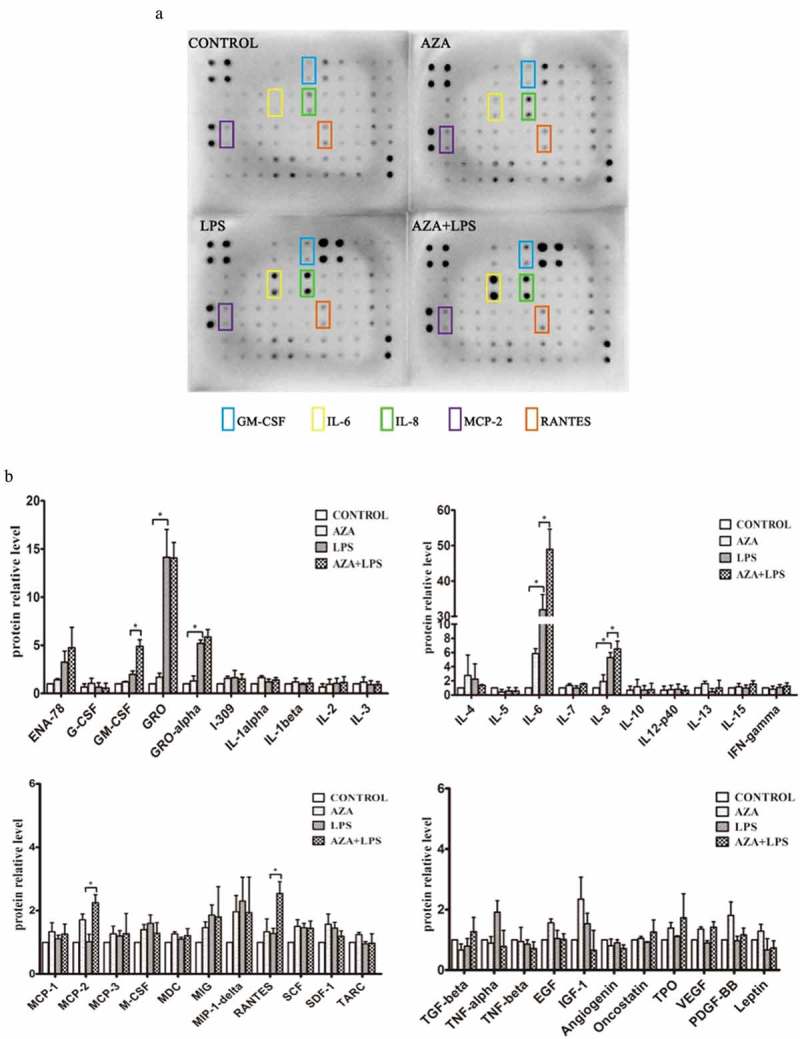
The effect of 5-Aza-CdR on the expression of inflammatory cytokines in hDPCs. (a) Cell culture media was collected from untreated hDPCs, 5-Aza-CdR-treated hDPCs, LPS-induced hDPCs, and 5-Aza-CdR-pretreated and LPS-induced hDPCs and subjected to human cytokine antibody arrays to assess the secretion of 42 cytokines. (b) The relative quantitative analysis of antibody arrays. The results are presented as means ± SD of three independent experiments; *P ＜0.05.

5-Aza-CdR is widely used as an epigenetic modulator to demonstrate DNA methylation. To determine whether DNA methylation is involved in inflammation of the dental pulp, LPS-induced hDPCs were pretreated with 5-Aza-CdR, and cytokine antibody arrays were used to examine the levels of 42 cytokines related to immunity and inflammation. 5-Aza-CdR alone was not able to induce significant expression of cytokines compared with the control group. However, 5-Aza-CdR pretreatment significantly increased the expression levels of IL-6, IL-8, GM-CSF, MCP-2 and RANTES compared with those observed in cells treated with LPS alone (*P* < 0.05). Among these cytokines, IL-6 and IL-8 were the most dramatically increased by LPS stimulation compared with their expression in the control and 5-Aza-CdR pretreatment groups (Figure 1(a, b)).

### 5-Aza-CdR enhanced the expression of IL-6 and IL-8 in LPS-induced hDPCs

**Figure 2.**
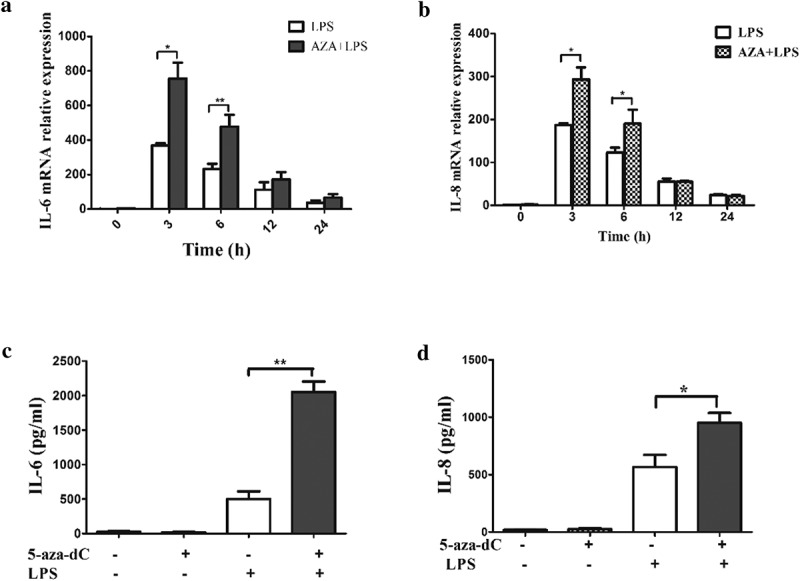
The differential expression of inflammatory cytokines induced by LPS in hDPCs with or without 5-Aza-CdR pretreatment. (a) Cells were collected from LPS-treated hDPCs with or without 5-Aza-CdR pretreatment. The mRNA expression of IL-6 was measured by qRT-PCR. (b) Cells were collected from LPS-treated hDPCs with or without 5-Aza-CdR pretreatment. The mRNA expression of IL-8 was measured by qRT-PCR. (c) Cell culture media were collected from LPS-treated hDPCs for 24 h with or without 5-Aza-CdR pretreatment. The protein expression level of IL-6 was measured by ELISA. (d) Cell culture media was collected from LPS-treated hDPCs for 24 h with or without 5-Aza-CdR pretreatment. The protein expression level of IL-8 was measured by ELISA. The results are presented as the mean ± SD of three independent experiments; *P ＜0.05; **P < 0.01.

To verify the results of the antibody arrays, the expression levels of IL-6 and IL-8 were measured by qRT-PCR. After 48 h of incubation with and without 5-Aza-CdR, the cells were stimulated with LPS for 0, 3, 6, 12 and 24 h. The mRNA levels of IL-6 and IL-8 were significantly increased beginning at 3 h in 5-Aza-CdR-pretreated cells relative to their levels in those stimulated by LPS alone (Figure 2(a, b)). Similarly, upregulation of IL-6 and IL-8 proteins was also observed using ELISA after pretreatment with 5-Aza-CdR in LPS-stimulated hDPCs (Figure 2(c, d)).

### 5-Aza-CdR upregulated NF-κB and MAPK signaling activity in LPS-induced hDPCs

**Figure 3.**
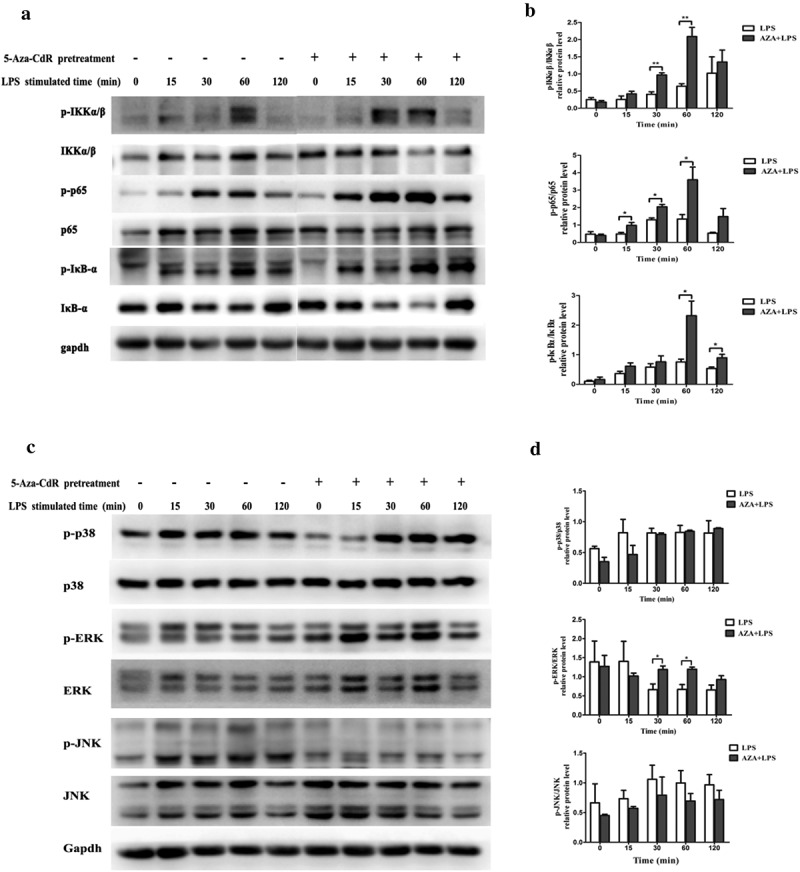
Effects of 5-Aza-CdR pretreatment on LPS-induced activation of the NF-κB and MAPK signaling pathways in hDPCs. Cells were pretreated with 10 µM/l 5-Aza-CdR for 48 h followed by stimulation with 1 µg/ml LPS. (a) The phosphorylation of IKKα/β, IκBα, and p65 in the NF-κB pathway was examined by western blot. GAPDH was used as an internal control. (b) The histogram shows the relative quantitative analysis of phosphorylation of IKKα/β, IκBα, and p65 in cells pretreated with 5-Aza-CdR compared to cells treated with LPS alone. (c) The phosphorylation of p38, ERK, and JNK in the MAPK pathway was examined by western blot. GAPDH was used as an internal control. (D) The histogram shows the relative quantitative analysis of the phosphorylation of p38, ERK, and JNK in cells pretreated with 5-Aza-CdR compared to that in cells treated with LPS alone. The results are presented as the mean ± SD of three independent experiments; *P ＜0.05; **P < 0.01.

NF-κB-mediated signal transduction is crucial for inflammatory cytokine production in response to LPS simulation. To determine the role of DNA methylation in the activation of the NF-κB pathway in LPS-stimulated hDPCs, phosphorylation of IKKα/β, IκBα, and p65 was analyzed by western blot. As shown in Figures 3(a and b), 5-Aza-CdR pretreatment remarkably enhanced the phosphorylation of IKKα/β, IκBα, and p65 compared with stimulation with LPS alone (*P* < 0.05).

MAPK signaling pathway is another important inflammation-related signal transduction. To determine the role of MAPKs in upregulating 5-Aza-CdR-induced cytokine expression, the phosphorylation of p38, ERK1/2 and JNK was also assessed in LPS-stimulated hDPCs after pretreatment with or without 5-Aza-CdR for the indicated time. The results showed that the phosphorylation of ERK increased in 5-Aza-CdR pretreatment group. Nevertheless, pretreatment with 5-Aza-CdR did not affect the phosphorylation of p38 and JNK (*P* > 0.05).

### 5-Aza-CdR reduced the 5mC level of the TRAF6 promoter

**Figure 4.**
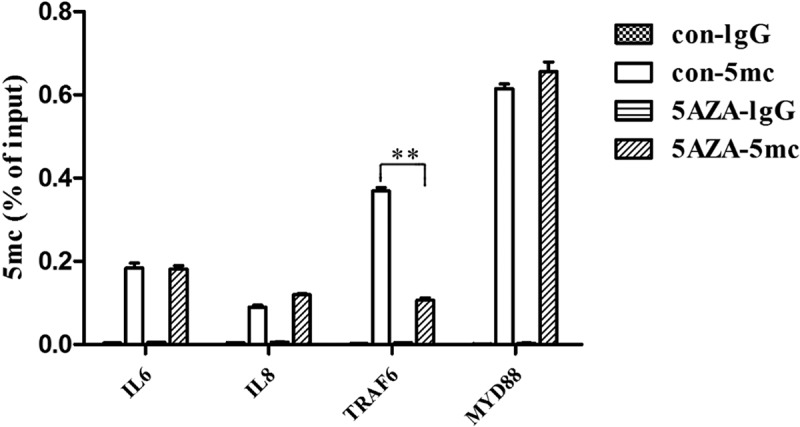
The effects of 5-Aza-CdR on the 5mC levels of the MyD88, TRAF6, IL-6 and IL-8 gene promoters in hDPCs. Dynamic methylation levels of the MyD88, TRAF6, IL-6 and IL-8 gene promoters were evaluated with MeDIP-PCR in the 5-Aza-CdR and control groups after 6 h of LPS stimulation. The results are presented as the mean ± SD of three independent experiments; *P < 0.05; **P < 0.01.

DNA methylation can modulate the development of inflammation by regulating the methylation status of both pro-inflammatory cytokines and signal transduction molecules [21,22]. MyD88 and TRAF6 are two canonical transduction molecules that play central roles in activating NF-κB and MAPK signaling pathways. To explore the mechanism of DNA methylation in the LPS-induced inflammatory response of hDPCs, the dynamic methylation status of the gene promoters of MyD88, TRAF6, IL-6 and IL-8 was examined using methylated DNA immunoprecipitation-PCR after 6 h of LPS stimulation in the 5-Aza-CdR and control groups. The results showed that the 5mC level of the TRAF6 promoter was significantly decreased in the LPS-induced hDPCs after 5-Aza-CdR pretreatment (Figure 4, *P* < 0.05). However, the 5mC levels of the IL-6, IL-8 and MyD88 promoters did not significantly change.

## Discussion

Bacterial infection is considered the most important etiological factor in pulpal and periapical diseases [23,24]. LPS from the cell walls of gram-negative bacteria can penetrate into the dental pulp and trigger inflammatory responses, which play a vital role in endodontic infection [25,26]. Cytokines and chemokines, such as IL-6 and IL-8, that are mediated though the NF-κB and/or MAPK signaling pathways are involved in LPS-induced inflammatory reactions of dental pulp cells [6,7,27]. In the present study, LPS was used to established inflammatory model of dental pulp, and it significantly upregulated the secretion of several cytokines, including IL-6 and IL-8, which is in agreement with the observations in LPS-treated dental pulp cells by Chang *et al.* [27].

DNA methylation is a key epigenetic modification in mammalian genomes that influences gene expression by altering the methylation levels of CpG sites. DNA methylation can transcriptionally induce pro-inflammatory cytokine production during the inflammatory reaction and has profound impacts on the development of inflammation-related diseases [12,13,28,29]. Demethylation treatment using 5-Aza-CdR followed by LPS stimulation reveals a significant enhancement of IL-6 expression in monocytes [30]. 5-Aza-CdR can reduce the methylation status at the IL-8 promoter and greatly enhance PGE2-mediated IL-8 mRNA expression in astrocytoma cells [31]. Cardoso et al. [10] showed that the presence of inflammation in human dental pulp is associated with the loss of DNA methylation in the promoter region of IFN-γ. In the present study, to investigate the role of DNA methylation in the inflammatory response of dental pulp, the effects of 5-Aza-CdR on cytokines and chemokines in LPS-treated hDPCs were investigated using a cytokine antibody array. The results showed that 5-Aza-CdR pretreatment significantly increased the expression levels of several cytokines, and the increases of IL-6 and IL-8 were particularly pronounced. 5-Aza-CdR significantly increased IL-6 and IL-8 levels in human bronchial epithelial cells, which support the observation in our study [32]. This pro-inflammatory effect of 5-Aza-CdR in LPS-treated hDPCs suggested that DNA methylation plays an important role in the progression of dental pulp inflammation.

The NF-κB and MAPK signaling pathways play crucial roles in mediating inflammatory response induced by LPS. DNA methylation can influence the inflammatory response by regulating the phosphorylation level of key factors in the NF-κB and/or MAPK signaling pathways. Demethylation treatment with 5-Aza-CdR elevates the phosphorylation of IKKα/β and IκB, which consequently activate the NF-κB signaling pathway and promote the transcription of target genes [33,34]. There were DNA methylation changes in 27 genes promoter of the MAPK pathway in human peripheral blood mononuclear cells and plasma samples from children with ambient exposure to air pollutants *in vivo* [35]. In contrast, a study of lung tissues reported that 5-aza-CdR significantly inhibited the phosphorylation of JNK, ERK and p38, which suppressed the activation of the LPS-induced MAPK signaling pathway [36]. In the present study, to investigate whether 5-Aza-CdR enhanced LPS-induced inflammatory cytokine production through activating signaling pathways, we assessed the activation of key signaling molecules of the NF-κB and MAPK pathways. The results indicated that 5-Aza-CdR pretreatment significantly enhanced the phosphorylation of IKKα/β, IκBα and p65 in the NF-κB pathway and the phosphorylation of ERK in the MAPK pathway. The activation of the NF-κB and MAPK signaling pathways can promote cytokine and chemokine release and result in the development of dental pulp inflammation [6,7]. The effect of 5-Aza-CdR on the activation of signaling pathways in LPS-stimulated hDPCs indicated that DNA methylation might regulate the inflammatory response of hDPCs by modulating the NF-κB and MAPK pathways.

DNA methylation epigenetically regulates inflammatory responses by several different mechanisms. It could directly mediate the transcription of pro-inflammatory cytokines, such as IL-6, IL-8, and TNF-α, by modulating the methylation level of the gene promoters [21,37]. DNA methylation is also involved in inflammation-related signaling pathways by epigenetically regulating the transcription of TLRs or signal transduction molecules, such as TLR2, MyD88, and TRAF6 [22,38]. MyD88 and TRAF6 are two canonical transduction molecules that play central roles in activating NF-κB and MAPK signaling pathways [39]. Recent studies have demonstrated that the dynamic methylation status of MyD88 and TRAF6, important intracellular signal transducers of the NF-κB and MAPK signal pathways, can affect the activation of downstream signaling pathways and is related to inflammatory diseases [38,40]. TRAF6 has been found to present hypermethylation in peripheral blood mononuclear cells in inflammatory bowel disease [38]. In the livers of rats administered ethanol, the methylation levels of TRAF6 and MyD88 significantly increased with increasing blood alcohol level after feeding the rats the major methyl donor SAMe [41,42]. To explore the molecular mechanism of DNA methylation in dental pulp inflammation, we analyzed the 5mC level in the gene promoters of MyD88, TRAF6, IL-6 and IL-8. Among these promoters, only the TRAF6 promoter showed a significant decrease in 5mC level after 5-Aza-CdR pretreatment in the LPS-induced hDPCs, suggesting that DNA methylation upregulated methylcytosine specifically at the TRAF6 gene promoter. These findings imply that DNA methylation might participate in the regulation of the LPS-induced inflammatory response by modulating the methylation status of TRAF6 promoter in hDPCs.

## Conclusions

This study demonstrated that 5-Aza-CdR can promote the development of inflammation induced by LPS by upregulating the expression of pro-inflammatory cytokines and activating the NF-κB and MAPK signaling pathways by decreasing the methylation level of the TRAF6 promoter in hDPCs. These findings indicate that DNA methylation plays an important role in the inflammation induced by LPS in hDPCs and may open new avenues for research into the treatment of dental pulp inflammation.

## Highlights

- 5-Aza-CdR significantly increased the LPS-induced pro-inflammatory cytokine expression in hDPCs.
- 5-Aza-CdR enhanced the phosphorylation of IKKα/β, IκBα, p65 and ERK in NF-κB and MAPK pathways in LPS-treated hDPCs.
- 5-Aza-CdR down-regulated the 5mC level of the TRAF6 promoter in LPS-stimulated hDPCs.
- DNA methylation plays an important role in the inflammation induced by LPS in hDPCs.
